# Going Abroad

**Published:** 1874-06

**Authors:** 


HALL'S

### JOURNAL OF HEALTH

AND

### MISCELLANY.

Vol. XXI.—JUNE, 1874.—No. VI.

### GOING ABROAD.

There is so much that is new, and strange, and waking up, in going abroad it is no wonder that multitudes regard it as one of the greatest of good fortunes to be able to "go to Europe." But many return without having their anticipations met, from want of proper information on what might be called minor matters.

Secure a good "berth" in time. Half the comfort of a sea voyage is often lost, and positive discomforts, almost unendurable, have to be met sometimes by being compelled to take what you can get in the way of sleeping accommodations. A first voyager is in his "berth" almost half the time—not a few scarcely leave it until they get on the other side; hence, it is of very great importance that there should be no leakage of water; that there should not be a water closet on the other side of an inch partition; that the propeller should not be at the head of your bed; that you should not be next door to the smoke stack, to keep you steamed day and night; that the pantry should not be your nearest neighbor, with its innumerable fumes and its constant clatter of crockery and wagging of servants' tongues; that you should not be at the bottom of the hatchway, to have a constant stream of persons passing your door. Do not take an inside room; let it have a window, or at least a porthole, which looks out upon the sea; but, as the porthole is closed during the voyage, it is greatly better to have a berth one story higher. A person of even moderate means had better pay twenty dollars to some one familiar with sea life to choose a berth.

Take about one third more money than you think necessary for travelling expenses; because you will be tempted to make unexpected purchases, and to deviate from your journey at certain points so as not to miss seeing places of interest which you did not know before you left were so near you. Don't go alone. There is no position so utterly woe begone as to be in a strange land and among a strange people and have no companion. Even in one's own country it is dreary enough; but, in having a friend with you, do not be placed in a condition to be controlled in your movements—to be compelled to go where you do not wish to, or to pass by localities which you are specially anxious to see. In one sense it is a great trouble to have a woman along—that is, if it is considered a trouble to show such attentions as they necessarily require; at the same time, if the woman is young and pretty, or if she be a lady of refinement and cultivation, with conversational ability, and has that quiet composure of deportment which marks well bred people, the pleasure of travel is just exactly doubled, as, besides the advantages of companionship, you have more attention in all public places, have better seats at table, have more commodious rooms at hotels, and more deference from servants, and clerks, and landlords, and are more likely to make desirable acquaintances.

If you have not plenty of money, stay at home until you do. Avoid placing yourself in a position which makes it necessary to debate the expenditure of every dollar, and be constantly under the necessity of making painful calculations of cost.

After three trips abroad, and making experiments, the unhesitating conclusion has been arrived at that it is most satisfactory to put up at the best hotels and travel in first class conveyances. It costs more, perhaps—more on an average—but then you have more room, more consideration and attention from officials and menials, and are very sure to avoid coming in contact with undesirable persons. Economies in eating and dress will more than make up this extra expense of fine hotels and first class tickets. It is not dress, but manners and deportment which command consideration and respect when travelling abroad; in fact, foreigners are rather inclined to a pitying contempt for fine dressed people in travelling conveyances; comfortable and substantial clothing, plain not loud, carries with it a letter of recommendation to the better class of Europeans.

An observant person will soon learn by experiment that he will feel better after a plain, substantial dinner costing half a dollar than a bill of fare at five, because he does not eat to bursting; does not eat so much as to make it an effort to rise from the table; as to make him avoid a cough for fear it may all come up again; and then feel too dull and stupid for the next hour or two to entertain himself or anybody else. The best system of eating while travelling is, first of all, regularity, then plain and nutritious food—food which has substance in it, and which imparts strength, and vigor, and elasticity, both of body and mind. Hence, they are wisest who take bread and butter and a piece of meat for breakfast, the same for dinner, adding one vegetable, and for tea (not later than an hour after sundown), a cracker or toast, broken into a cup of English breakfast tea—not coffee. The drink at breakfast and dinner should be something warm. Considering that gormandizing unfits one for the enjoyment of travel, and often occasions inconvenient attacks of sickness on public conveyances and at night, common sense indicates a confinement to plain, nutritious, and, as a matter of course, inexpensive food.

By all means avoid binding yourself a month or more beforehand to take passage for home at a specified day; it will be a weight and an anxiety all the time, an impending something akin to a nightmare.

A much better plan is to leave yourself free to start for home when it suits you. This prevents a feeling of hurry and its consequent worry, causing you to give much less time to seeing places and things than they merit, and to pass by others of great interest, to be a permanent regret after you get home again.

Never take a berth for your return home, unless you are familiar with the ship, except at the port of embarkation—you will be certainly cheated. The reserve berths at Paris, or elsewhere, on Liverpool steamers are purposely the worst on the ship, although they will always tell you they are the very best. If at Liverpool you can see for yourself; if at a distance you must take the word of an agent, who may, all over the world, be considered a know nothing. There is no one thing, perhaps, in connection with foreign travel about which so many unblushing falsehoods are told as about all the berths being engaged beforehand, even for months. It is at least nine hundred and twenty-six times nearer the truth that not one steamer in fifty ever leaves port without one or more berths unoccupied. Cases are known where the very best berths at the last day have been disposed of at the minimum price. Hence, it is safer to take your chances and go to Liverpool and wait for a good berth, amusing yourself with excursions to Wales, the slate quarries at Bangor, and quaint old Chester with its wealth of memories. It is better to see one town well, and know all about it, than visit a dozen in a hurry, and when you return home be only able to say of them they were beautiful, splendid, magnificent.

Thirty years ago a party of ladies and gentlemen came to Quebec late at night, and on being invited to go to the Plains of Abraham, the spot where Wolfe fell, and the Indian village, they replied that they had not time—they only came there "to have it said" they had been to Quebec. "To have it said" is the highest ambition of quite a number of more modern travellers.

When you put up at a hotel and design remaining over two days, have your bill sent to you at the end ef every twenty-four hours, so as to have an idea of their rates of charges, thus lessening the chances of having an account made out against you a mile longer than you anticipated, with the consequent annoyance connected with it. It is a very commendable caution at the Swiss hotels, in some parts of Germany and in the large cities of Austria to have your bill sent to your room every day at noon—not that you are expected to pay it, but that you may know at what rate you are living, and may correct any charges improperly placed in your account. Always insist on having your bill made out four or five hours before the time of leaving, so as to give full leisure to have mistakes corrected.

The rates of passage to Europe this season, with first class berths, are from seventy dollars in paper to a hundred and twenty-five in gold. Twenty or thirty dollars, or more, may be saved by paying for a return ticket in any ship of the line, and at any time within a year. One prime advantage in this arrangement is that your journey home is paid for and thus not find yourself in Liverpool without having money enough to secure a berth, imposing the necessity and the expense of "cabling" for funds, or the humiliation of borrowing from friends, or soliciting aid from strangers, or almost going on your knees to the American Consul or steamer agents. Such things occur every day in the year at Liverpool.

Last year two ladies applied to the proprietors of a line of steamers, not only to give them passage to New York, but to furnish them with money to redeem their trunks, which had been left in Paris, in pledge for hotel bills. Their father's check was good for a hundred thousand dollars. They knew the perfect safety of the transaction, but the owner of the line did not; and, from principle, he refused, and very properly. Anybody could tell the same tale. Henry Ward Beecher is reported to have said one day that he knew rich men in Brooklyn who would tell the collectors on ferry boats that they were commuters, when they were not, thus saving two cents; much more might one lie to get a passage to New York. As the parents of these ladies were summering out West a cable message could not reach them, so they had to wait for remittances by mail. But how often does it happen that persons away from home and in strange places, wait for remittances; so wearily wait, and remittances don't come. Almost any hotel proprietor in the large cities could tell of cases of this sort by the month.

Last year a gentleman, an officer in the late war, found himself and wife in London, without funds to pay their passage home from that port. They had been at a first class hotel, but as their means became more scant they had gone to less expensive quarters, down, down, down, until at the time spoken of they occupied a hired room; the wife was sick; one article and another of clothing had been pawned to procure means for food and medicine. The "water proof," the shawl, the traveling bag, the umbrella, the parasol, and among the list, right in sight, was a night cap and chemise. There was a plate of grapes on the table, and the husband was invited to help himself. He declined, but said they would do his wife so much good, and if there was no objection, he preferred to take them to her, if a paper could be had to wrap them up in.

Moral.—If you don't want to be hunted up by some needy American, of whom you never heard, do all you can to prevent your name and place of stopping being put in the "American Register," and it will take a long time to find you out. One of the proprietors of a line of steamers to New York, said last summer, in Liverpool, that in not one single instance of helping others to New York, in whole or part passage money, had he been repaid, and, after one case of great aggravation, he swore he would never give aid in that way again to the amount of one single dollar.

Hence, if your means are limited, pay for your return passage before you leave New York, and leave your voucher for the same in Liverpool, as you might lose it in your travels over the Continent.

